# Marine Algal Antioxidants

**DOI:** 10.3390/antiox9030206

**Published:** 2020-03-02

**Authors:** Clementina Sansone, Christophe Brunet

**Affiliations:** Stazione Zoologica Anton Dohrn, Villa comunale, 80121 Napoli, Italy; christophe.brunet@szn.it

Sea and marine biodiversity exploration represents a new frontier for the discovery of new natural products with human health benefits (“the exploitable biology”, [1]).

New compounds suitable for nutraceuticals, cosmeceuticals, or pharmaceuticals require (i) eco-friendly production and (ii) bioactivity against illness, thus making microorganisms potential interesting targets. Among them, microalgae provide advantages as they are photosynthetic, high growth rate organisms that are easy to cultivate and require less space than higher plants, together with displaying high chemodiversity—though this has barely been explored—coupled with high biodiversity [2]. Although very attractive compared to higher plants, microalgae biotechnology still requires further research and development to lower its cost and enhance practical and industrial interest [3,4]. One of the main branches of the biotechnological exploration of marine algae concerns bioactive and, especially, antioxidant compounds.

This Special Issue, concerning marine algal antioxidants, contains eleven contributions detailing recent advances in this field; experimental results and technical improvements are presented and discussed.

Antioxidant bioactivity concerns different families of compounds, but this issue is focused on the microalgae richness of such compounds (e.g., [5,6]). Among the huge variety of antioxidant compounds, algae derived carotenoids are the most well known, together with other bioactive compounds, such as polyphenols, sterols, carbohydrates, and vitamins [6]. The synergistic effect of all of these families in unicellular organisms induces the high antioxidant power of microalgae that is comparable, or even higher than, the antioxidant activity of higher plants or fruits [5]. The potentiality of a single microalgae cell compared with that of a multicellular plant presents a biotechnological challenge for developing microalgae as an efficient and ecosustainable “bio factory” of bioactive molecules with antioxidant activity. For this reason, it is very important to invest in research programs aiming to investigate the diversity of bioactive molecules along the microalgal biodiversity scale and its intracellular modulation [2].

In a recent study [7], the coastal diatom *Skeletonema marinoi* was used to investigate the modulation of lipophilic antioxidant compounds and the hydrophilic vitamin c by light manipulation. The results revealed a significant effect of light (intensity and/or distribution) on the production of antioxidants as well as a strong link between carotenoid operating photoprotection and the antioxidant molecules and activity modulation. This study confirms the role of light manipulation as a powerful tool for modulating the synthesis of antioxidant compounds in microalgae.

The most frequently investigated algae compounds are carotenoids due to their well-known bioactivity and human wellness benefits as well as their plasticity which allows them to be enhanced through abiotic factors, for example, light modulation in microalgae [2,8].

*Dunaliella salina*, a chlorophyte that is mostly used for biotechnological investigations and applications, mainly relies on the production of β-carotene [9], has been used as a model to study the modulation of carotenoids and β-carotene concentration with respect to the light spectrum [10,11]. This study demonstrates that monochromatic red light strongly affects the carotenoid pool, enhancing the β-carotene concentration as well as modifying the ratio between the different forms of β-carotene towards 9-cis β-carotene. These studies confirm the relevant role of light in shaping the carotenoid profile in microalgae, demonstrating that its modulation is of great interest for the biotechnological production of such bioactive compounds.

The enhancement of carotenoid production in algae can use genetic engineering and biomanipulation. In order to reach this goal, it is necessary to increase the knowledge about the biosynthetic pathways of these compounds as well as the modulation factors affecting the gene expression involved. Two brown algae, *Saccharina japonica* and *Cladosiphon okamuranus*, have been investigated thanks to the analysis of Genome–Scale Metabolic Networks (GSMNs, [12]). The authors were able to reconstruct the biosynthetic pathways of the main carotenoids in these two algae, highlighting the interest and scientific richness of such approach for the study of targeted biochemical pathways.

Together with carotenoids, chlorophylls and their derivatives are also of interest for biotechnological applications [13]. Enhancing the production of chlorophylls per biomass unit in microalgae and understanding the biosynthetic and degradation pathways of such molecules is therefore biotechnologically relevant. The study by Maroneze and collaborators [13] reported and discussed the modulation of the chlorophyll and carotenoid contents in the model species *Scenedesmus obliquus* with respect to the growth phase and the presence/absence of light, turning growth from autotrophy to heterotrophy. The authors demonstrated that the content and chemical forms of these compounds are affected by growth conditions, laying the foundation for up-scaling and massive production for industrial application.

On the other hand, i.e., in brown algae, fucoxanthin is now being investigated for its potential activity related to human health protection [14]. This pigment might be extracted from numerous classes of microalgae, including diatoms as well as brown macroalgae [14]. The anti-inflammatory, antioxidant, and antiproliferative effects of fucoxanthin were investigated on blood mononuclear cells and different cell lines [15]. The results clearly displayed the antiproliferative and antioxidant activities of fucoxanthin in vitro, highlighting the great interest in its potential use in nutraceuticals.

Pigments are not the only compounds presenting antioxidant properties; other lipophilic compounds such as phenols and hydrophilic compounds accompany them.

It is therefore of interest to investigate the best solvents for obtaining the best yield from the extraction of bioactive compounds from algal biomass. For this reason, in three brown algae [16], the antioxidant properties and antioxidant compound concentration were compared between seven extraction solvents or mixtures between them. This work defined the best extraction procedure for enhancing the harvesting of phenols, flavonoids, carotenoids, and chlorophylls. In the same framework, a technical approach comparing methodologies for the quantification of polyphenols was undertaken on the macroalga *Ulva intestinalis* [17], highlighting some uncertainties and difficulties in actual methodologies that require further optimization of the extraction, identification, and quantification of polyphenols.

In addition, polysaccharides are also relevant antioxidant compounds, and algae might be a relevant source for their production and use as nutraceuticals [18]. In the contribution by Le et al. [19], the authors compared data from different extraction procedures of the green alga *Ulva pertusa* in terms of antioxidant activity together with polysaccharide and ulvan contents. The differences between the various extracts were compared with regard to operational parameters such as power, time, water-to-raw-material ratio, and pH, in order to optimize the quantity yield of ulvan.

Last, but not least, extracts from *Fucus* spiralis and *Chlorella vulgaris* were tested as enhancers of the removal of heavy metals (Hg^++^, Ag, Sn, Pb) in patients with long-term dental titanium implants and amalgam filling restoration [20]. The authors demonstrated that long-term effects from nutritional supplementation with these algae result in the enhancement of heavy metal removal.

All of these contributions highlight the great potential of marine algae to provide substances/extracts that are able to protect or increase human wellness, and the need for optimization and technological/technical/scientific improvement to increase the biomass-harvesting efficacy with reduction of the production cost. Marine biotechnology relies on the exploration, discovery, and exploitation of marine algal species and/or products still requires research dealing with biodiversity (searching for new targeted species with peculiar biochemical profiles, for instance), chemodiversity (richness and diversity of bioactive molecule screening), bioactivity (antioxidant ability of the algal extracts), technological cultivation improvement (lowering the costs, co-cultivation, environmental modulation), and optimization of the extraction techniques. These steps are crucial to achieve the challenges offered by the green (blue in case of marine) biotechnological revolution which, in our point of view, cannot exist without deployment of the industrial use of (micro)algae.
